# Assessing Retail Fruit and Vegetable Availability in Urban and Rural Underserved Communities

**Published:** 2008-09-15

**Authors:** Akiko S Hosler, Deepa T Rajulu, Adrienne E Ronsani, Bonnie L Fredrick

**Affiliations:** Department of Epidemiology and Biostatistics, University at Albany School of Public Health; Bureau of Chronic Disease Epidemiology and Surveillance, New York State Department of Health, Albany, New York; Bureau of Chronic Disease Epidemiology and Surveillance, New York State Department of Health, Albany, New York; Wadsworth Center Genetic Services, New York State Department of Health, Albany, New York

## Abstract

**Introduction:**

Fruits and vegetables (F&Vs) are important parts of a healthy, balanced diet. Consumption of F&Vs is low among residents of socioeconomically disadvantaged communities. We investigated and compared retail F&V availability in urban and rural underserved communities in New York State.

**Methods:**

All food retail stores and farmers' markets (N = 263) in downtown Albany and in Columbia and Greene counties in New York State were visited and surveyed. Food stores were classified as *F&V stores* if they stocked more than the minimum varieties of fresh F&Vs defined by this study and as *fruit-for-snack stores* if they had ready-to-eat fruits only. Store density per 10,000 residents was calculated as a standardized measure of F&V availability. Adjustment weights were created to incorporate store size and business hours into the analysis.

**Results:**

The weight-adjusted density (per 10,000 residents) of F&V stores was 4.6 in Albany's minority neighborhood (reference category), 11.4 in Albany's racially mixed neighborhood (*P* = .01), 7.8 in Columbia and Greene counties' rural community (*P* = .10), and 9.8 in Columbia and Greene counties' small-town community (*P* = .02). Significant differences were not found in fruit-for-snack stores, which ranged from 2.0 per 10,000 in the mixed neighborhood to 3.4 per 10,000 in the rural community.

**Conclusion:**

The urban minority neighborhood had the most barriers to fresh F&Vs in retail outlets, even when compared with the rural community. The low availability of retail F&Vs in the minority neighborhood was attributed to the lack of supermarkets and not the absolute lack of food stores. Public health intervention strategies to increase retail F&V availability in urban minority neighborhoods may aid in mitigating these disparities.

## Introduction

Fruits and vegetables (F&Vs) are important parts of a healthy, balanced diet. Many studies have documented the benefits of F&V consumption for weight control (1,2) and prevention of chronic diseases, including cardiovascular disease (3,4), diabetes (5,6), and certain types of cancer (7). On the basis of recommendations by the *Dietary Guidelines for Americans* (8), *Healthy People 2010* (*HP 2010*) has set national objectives for increasing proportions of Americans who consume at least 2 daily servings of fruit and 3 daily servings of vegetables, with at least 1 serving being a dark-green or orange-colored vegetable (9). Yet most Americans do not consume the daily recommended amount of F&Vs (10,11). Consumption of F&Vs is reportedly low among socioeconomically disadvantaged, rural, and poor residents (12).

Reasons that people do not consume the recommended daily servings of F&Vs are complex. Environmental barriers, notably the limited availability of fresh produce in local retail stores, can be a factor (12-14). Studies have found that individuals living in close proximity to supermarkets were more likely to consume F&Vs than were people living in neighborhoods without supermarkets or located farther away from supermarkets (15-17). The inverse association between distance to the nearest supermarket and F&V consumption appears to be pronounced among African Americans (15) and low-income individuals (16). One study found that urban African American women who shopped at their neighborhood small-food stores consumed fewer F&Vs than did their higher-income peers who were able to shop in suburban supermarkets (18).

Studies suggest that consumption of F&Vs by disadvantaged individuals can be increased if they have access to a supermarket or food store that provides an adequate amount of affordable fresh produce (13,19,20). However, during the last few decades spatial disparities in access to retail fresh F&Vs have increased considerably (21). Supermarkets and grocery stores with produce departments are much less likely to be found in economically disadvantaged minority neighborhoods than in middle-class or affluent white neighborhoods (21-27). Residents in remote, rural communities also face barriers to obtaining retail fresh F&Vs (12).

We measured, quantified, and compared retail availability of fresh F&Vs in selected urban and rural communities in New York State. We developed a field survey tool; a store classification scheme; and a standardized, weight-adjusted method to calculate store density as a measure for fresh F&V retail availability. Our goals were to identify communities with barriers to obtaining fresh produce and to suggest interventions for improving retail availability of fresh F&Vs.

## Methods

### Study communities

The study communities were Columbia County, Greene County, and a downtown portion of the city of Albany, the designated underserved intervention communities for the Albany Prevention Research Center's Diabetes Prevention and Control Project (28). We defined downtown Albany ("Albany" hereafter) as 4 geographically contiguous zip code areas (12202, 12206, 12207, and 12210). An analysis of 2000 US census data showed that these areas formed a region with a population of low socioeconomic status within the city of Albany (29). Our previous study showed that residential racial composition was the sole predictor for availability of low-fat milk and high-fiber bread in these same communities (29). We divided Albany into a minority neighborhood, which was defined by census block groups (CBGs) with at least 50% nonwhite and/or Hispanic populations, and a mixed neighborhood, which was defined by CBGs with  less than 50% nonwhite and/or Hispanic populations.

We divided Columbia and Greene counties, where the population is predominantly non-Hispanic white, into a rural community and a small-town community. The rural community was composed of CBGs with populations that are 100% rural, as defined by the US Census Bureau (30), and the small-town community was composed of the two counties' remaining CBGs. This division contrasted sparsely populated rural regions with more populous small towns along the Hudson River.

### Data collection

**Figure 1.**
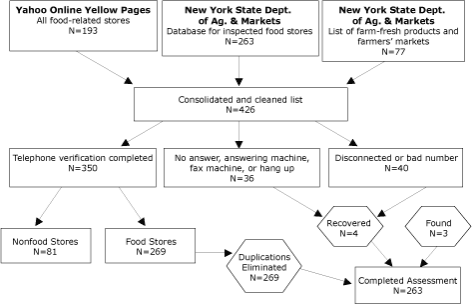
Flow Chart Depicting How Food Stores Were Identified, Albany, Columbia County, and Greene County, New York, 2003.

We visited and surveyed all food stores in the study communities during July through September 2003. We defined a “food store” as a retail store that was open for business for at least 5 days per week during the time of the survey and that stocked at least 1 of the following food items: milk, bread, or fresh produce. Farmers’ markets were included regardless of the number of days they were open each week. We used 3 sources to obtain a list of food stores: 1) the New York State Department of Agriculture and Markets’ (NYSDAM’s) current inspected food store database (accessed using a Freedom of Information Act request), 2) the NYSDAM’s online guide for farm-fresh products and farmers’ markets (31), and 3) online local yellow pages. We initially identified 426 potential food stores, including 7 farmers’ markets. We contacted all of the food stores by phone to verify whether they fit our food store definition and whether they were located in the study area. After eliminating nonqualified, closed, and duplicate stores, 263 stores remained and were surveyed. A detailed process of identifying food stores is shown in Figure 1.

**Table:** 

This flowchart depicts a series of boxes that explain the process of selecting eligible food stores. These boxes read from top to bottom and are connected with downward-pointing arrows. The first 3 boxes are arranged horizontally and contain text explaining the 3 sources from which food stores in New York state were chosen: 1) Yahoo Online Yellow Pages (all food-related stores, n = 193), 2) New York State Department of Agriculture and Markets (database for inspected food stores, n = 263), and 3) New York State Department of Agriculture and Markets (list of farm fresh products and farmers’ markets, n = 77). All 3 of these boxes have an arrow pointing downward to 1 box, which explains the next step in the process: “Consolidated and cleaned list (n = 426).”
The box describing the list derived from the 3 sources has 3 arrows that point downward to 3 separate boxes that are arranged horizontally and that describe the next phase in the process of identifying food stores: telephoning the stores. The first box says, “Telephone verification completed (n = 350).” This box is connected with 2 downward-pointing arrows to 2 boxes underneath it. The first box says, “Nonfood stores (n = 81).” The second box says, “Food stores (n = 269).” The “Food stores” box is connected with a right-pointing arrow to a box that says, “Duplications Eliminated (n = 256).” This box is connected with a right-pointing arrow to a final box that says, “Completed Assessment (n = 263.)”
The second box says, “No answer, answering machine, fax machine, or hang up (n = 36).” The third box says, “Disconnected or bad number (n = 40).” The second and third boxes have downward-pointing arrows that connect them to a box that says “Recovered (n = 4).” Next to this box is a stand-alone box that says, “Found (n = 3).” The boxes that say “Recovered” and “Found” are each connected with downward-pointing arrows to the final box that says, “Completed Assessment (n = 263).”

The 1-page survey assessed more than 10 types of food and nonfood items. Among other indicators, F&V presence and variety were assessed. A team of 2 or 3 trained surveyors conducted the in-store surveys. If fewer than 10 types of fresh F&Vs were sold, surveyors wrote down each type of F&V; if more than 10 types of F&Vs were sold, surveyors noted that there was a large variety. Surveyors noted whether at least 1 dark-green or orange-colored vegetable was present, and they collected information about store hours and number of cash registers used. We used the number of cash registers as a measure for store size, as suggested by a previous study (25). To ensure interrater reliability, results of the assessment were recorded when all members of the survey team agreed on the assessment decisions. As a standardized protocol, surveyors asked permission to conduct a survey, and all stores granted permission. Written consent was obtained from an employee of each store who was authorized to make customer-related decisions.

The location of each store was measured at the front door by coordinates using a hand-held global positioning system (GPS) device (Thales Navigation, Inc, Alexandria, Virginia). Survey data were analyzed using SPSS version 12.0 (SPSS, Inc, Chicago, Illinois), and GPS information was processed using MapInfo software (Pitney Bowes Software, Inc, Troy, New York).

### Measurement

**Figure 2.**
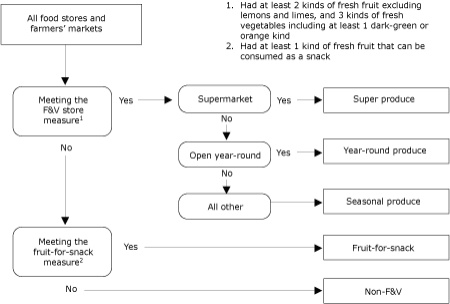
Flow Chart Depicting How Food Stores Were Classified, Albany, Columbia County, and Greene County, New York, 2003.

We initially grouped surveyed food stores using a business-type classification system developed by Morland et al (22). However, we found that considerable variations in fresh F&V presence and variety existed within the same business type. For instance, 46% of gas station stores had some fresh F&Vs, but 54% did not carry F&Vs at all. Among convenience stores in the same chain, some locations had produce sections but others did not. Furthermore, "supermarket" did not automatically mean that the store had a produce department; 1 large national chain's local stores had no produce departments at all. Therefore, we devised a store classification system to properly categorize the stores by the availability and variety of fresh F&Vs sold (Figure 2).

**Table:** 

This flowchart depicts the process of how food stores were classified. The flowchart contains a series of boxes that are connected to one another with downward-pointing or right-pointing arrows and arranged so that they read from top to bottom and left to right. Stand-alone text in the top right corner of the figure reads as follows: “1) Had at least 2 kinds of fresh fruit, excluding lemons and limes, and 3 kinds of fresh vegetables, including at least 1 dark-green or orange kind; and 2) Had at least 1 kind of fresh fruit that can be consumed as a snack.” These 2 numbered sentences are references used in the figure.
The first box of the flowchart is situated in the top left corner of the figure and says, “All food stores and farmers’ markets.” This box is connected by a downward-pointing arrow to a box underneath it that says, “Meeting the fruit and vegetable store measure1.” This box is connected to 2 boxes, 1 that says “Yes” and the other that says “No.” The “Yes” box is connected with a right-pointing arrow to a box that says, “Supermarket.” The “No” box is connected with a downward-pointing arrow to a box that says, “Meeting the fruit-for-snack measure^2^.”
The “Supermarket” box is connected to 2 boxes, 1 that says “Yes” and the other that says “No.” The “Yes” box is connected with a right-pointing arrow to a box that says, “Super produce.” The “No” box is connected with a downward-pointing arrow to a box that says, “Open year-round.” The “Open year-round box” is connected to 2 boxes, 1 that says “Yes”; and the other that says “No.” The “Yes” box is connected with a right-pointing arrow to a box that says, “Year-round produce.” The “No” box is connected with a downward-pointing arrow to a box that says, “All other.”

We categorized the business as an *F&V* *store* if it stocked at least 2 types of fresh fruit, excluding lemons and limes, and at least 3 types of fresh vegetables, including at least 1 dark-green or orange-colored vegetable. We further grouped the F&V stores into 3 types: *super produce stores*, or supermarkets with a produce department; *year-round produce stores*, which were nonsupermarket F&V stores that operate year-round, including grocery stores, convenience stores, and specialty food stores; and *seasonal produce stores*. The last category included seasonal farm (ie, barn) stores, roadside huts and stands, and farmers' markets. Stores that did not meet the F&V measure but did carry at least 1 type of ready-to-eat fresh fruit, such as apples, oranges, and bananas, were designated as *fruit-for-snack stores*. We determined that recognition of this type of store would be useful for promoting fruit consumption (ie, choosing a piece of fruit in lieu of candy or a snack food). The remaining stores were grouped into the *non-F&V store* category.

### Data analysis

As a standardized proximal measure of fresh F&V availability, we calculated store density per 10,000 residents and compared the densities of the 4 communities. Data from the 2000 census were used to determine total number of residents. A simple store density calculation would treat all stores equally, including large supermarkets, small corner stores, and farmers' markets that open only 4 to 7 hours per week. To adjust for store size and operating hours, we devised a simple adjustment weight. The number of cash registers, a surrogate measure for store size (25), was natural-log–transformed and added the constant of 1 to normalize the right-skewed distribution with the minimum value of 1. Store hours were measured by per-week operating hours. Store hours were normally distributed, with 98 hours as both the mean and the median values. A 98-hours-per-week operation is equivalent to opening from 7:00 a.m. to 9:00 p.m. daily, a time period we determined was adequate for most residents to shop for F&Vs. We designated a weight of 1 for a store with 1 cash register that was open for 98 hours per week. The weight increased if the store had more than 1 cash register and decreased if the store operated fewer than 98 hours per week.

Therefore, the adjustment weight (w*i*) was obtained from 1 plus the natural-log–transformed number of cash registers multiplied by the store hours per week divided by 98. For stores open 98 hours per week or longer, a constant of 98 was used:

w*i* = (1+ nlog c*i* )(h*i* / 98) where w*i* = adjustment weight, c*i* = number of cash registers, and h*i* = business hours per week if h*i* <98, h*i* = 98 for all other stores.

In aggregate, the adjustment weight amplifies the effects of super produce stores almost 3 times but reduces the effects of seasonal produce stores by half. Other types of stores remain mostly unchanged.

We compared unadjusted and weight-adjusted store density per 10,000 residents of the study communities, using the minority neighborhood in Albany as the reference category. The *z* score was used for statistical analysis of significance. Significance was determined at an α level of .05. The institutional review board of the New York State Department of Health reviewed and approved the study protocol.

## Results

Characteristics of the study communities are presented in Table 1. The minority neighborhood had the highest percentage of racial and ethnic minorities (74.1%) of the 4 communities, and the rural community had the lowest percentage (4.6%). One in 3 residents in the minority neighborhood and 1 in 4 residents in the mixed neighborhood were living below poverty. In both the mixed and minority neighborhoods, three-quarters of housing units were renter-occupied. At least one-third of households in both the minority and mixed neighborhoods did not have a vehicle. The rural community, with a total population of nearly 62,000, had the lowest rates among the 4 study communities of racial/ethnic minorities (4.6%), individuals living in poverty (8.9%), renter-occupied housing (24.6%), and households without a vehicle (5.9%), and it had the highest rate of citizens aged 65 years or older (16.3%).

A total of 263 eligible food stores and farmers' markets were categorized with our classification system. Of stores designated as F&V stores, which made up 36.5% of all food stores, 16 were super produce stores, 54 were year-round produce stores, and 26 were seasonal produce stores. Most F&V stores stocked far more than the minimum fresh F&Vs defined by this study. Additionally, 37 stores (14%) were fruit-for-snack stores, and 130 stores (49%) were non-F&V stores. Seventeen stores classified as non-F&V stores carried some lemons, limes, and/or vegetables, but none were dark green or orange-colored. A summary of statistics for the numbers of cash registers and store hours, as well as adjustment weights, is shown in Table 2.

The availability of fresh F&Vs was measured by store density (Table 3). Unadjusted population density of F&V stores was 4.2 per 10,000 residents for the minority neighborhood, 7.3 per 10,000 residents for the mixed neighborhood, 6.5 per 10,000 residents for the rural community, and 6.8 per 10,000 residents for the small-town community. No statistically significant differences were found among the communities.

Population densities of all stores were weight-adjusted to factor in store size and business hours. For every 10,000 residents the communities had the following number of F&V stores: 4.6 for the minority neighborhood, 11.4 for the mixed neighborhood, 7.8 for the rural community, and 9.8 for the small-town community. The store densities were significantly higher for the mixed neighborhood (*P* = .01) and the small-town community (*P* = .02) than for the minority neighborhood. This was largely because the adjustment weight upwardly corrected the contribution of super produce stores, which were present in the mixed neighborhood (7.3 per 10,000 residents), rural community (2.4 per 10,000 residents), and small-town community (4.0 per 10,000 residents) but not in the minority neighborhood. The contribution of seasonal stores was downwardly corrected by the weight, primarily because of their shorter business hours, but the small-town community still had a significantly higher density of seasonal stores (1.6 per 10,000 residents, *P* = .04) than did the minority neighborhood, where there were no seasonal stores. No significant differences were found among year-round produce stores (ranged from 4.0 per 10,000 residents in the mixed neighborhood to 4.6 in the minority neighborhood), fruit-for-snack stores (ranged from 2.0 per 10,000 residents in the mixed neighborhood to 3.4 in the rural community), and all food stores combined (ranged from 17.7 per 10,000 residents in the rural community to 25.4 per in the mixed community).

## Discussion

Our study demonstrated that the urban minority neighborhood was the most disadvantaged in terms of retail F&V availability, as measured by the population density of F&V stores. The low retail availability of fresh F&Vs in this community appears to be largely because of the lack of super produce stores and not the absolute lack of food stores. In fact, the urban minority community had a large number of food stores, and, of the 4 communities, the highest population density of year-round produce stores and non-F&V stores and the second-highest population density of fruit-for-snack stores in the weight-adjusted model. This is a case of relative deficiency, in which high-impact super produce stores were conspicuously missing. In contrast, the urban mixed neighborhood had 3 large super produce stores. The higher density by population of supermarkets and large grocery stores in racially mixed areas compared with predominantly minority areas in the urban setting has been reported by other studies conducted elsewhere in the United States (22,26,27).

Although the results were not statistically significant, the rural community had higher overall fresh F&V availability compared with the urban minority neighborhood. The rural community had the lowest density by population of total food stores but had the highest density of fruit-for-snack stores and the second highest density of year-round produce stores. The high availability of ready-to-eat fruits can be explained by the existence of many family-owned fruit orchards and berry farms in this community. The small-town community had the second highest overall F&V availability, with a balanced representation of super produce, year-round produce, and seasonal produce stores. In the winter when seasonal produce stores are not in business, both communities in Columbia and Greene counties probably see some decline in F&V availability. However, the decline probably does not reach the level of the urban minority neighborhood because these seasonal stores make a small contribution to the overall F&V availability in the rural and small-town communities.

Our study has several limitations. We used a cross-sectional survey of food stores and did not adjust for the variability caused by harvest, delivery, or reshelving schedule. We did not directly assess quantity or quality of fresh F&Vs because of a lack of standard measurements suited for field studies. The designation of neighborhoods and communities was made on the basis of CBG data and may differ from actual areas designated as communities. The rural community encompasses a large tract of land, and most remote portions of the community may have more environmental barriers to retail F&V availability. The store classification system and the adjustment weight were new tools and have not been tested in other communities. This study, by design, did not assess the availability of non-retail fresh F&Vs such as those grown in private or community gardens. Availability of canned, jarred, and frozen F&Vs was not assessed because of time constraints, the lack of standard measurements, and the presence of salt, sugar, and nutrient loss that can occur in the process of canning, jarring, and freezing F&Vs.

A growing need exists to develop public health policies and innovative intervention strategies to increase retail availability of fresh F&Vs in disadvantaged communities. To do so, scientific yet simple field methods to measure retail food availability need to be established that can be applied to both urban and rural communities. The "food deserts" controversy in the United Kingdom revealed that a few small-scale exploratory studies of retail food availability in poor urban neighborhoods were used repeatedly — and in some instances were misinterpreted — to form food and nutrition policies (32). This incident highlights the importance of applying sound science in community food assessment for policy recommendations.

Because very few existing food availability studies directly compared rural communities with urban communities, findings from this study will be useful for making policy recommendations and planning interventions from a global perspective. This study suggests that urban minority neighborhoods should be a priority for improving retail fresh F&V availability. Although bringing new retail produce stores into an urban environment is not an easy task, 1 study explains that collaboration among stakeholders, including community leaders, business owners, growers, media, and local government, can greatly improve the chances of successful introduction and retention of the stores (23). Working with the existing fruit-for-snack stores to add more F&Vs to their inventory is another intervention idea. In-store promotion and nutrition education, such as taste testing, giveaways, and cooking demonstrations to increase local consumer demand, should accompany this effort.

## Figures and Tables

**Table 1:** Selected Characteristics of Study Communities, Albany, Columbia County, and Greene County, New York, 2003

Characteristic	Minority Neighborhood^a^	Mixed Neighborhood^b^	Rural Community^c^	Small-Town Community^d^
Total population	26,045	14,969	61,652	49,637
Racial/ethnic minorities, %	74.1	32.2	4.6	16.4
Individuals aged ≥65 y, %	10.6	12.2	16.3	15.9
Individuals living below poverty, %	33.3	25.3	8.9	12.4
Renter-occupied housing, %	75.4	75.2	24.6	34.2
Households without a vehicle, %	43.3	33.0	5.9	11.2
Total no. of food stores	53	29	94	87

Abbreviation: CBGs, census block groups.

a Region within the city of Albany containing CBGs composed of ≥50% nonwhite and/or Hispanic populations.

b Region within the city of Albany containing CBGs composed of <50% nonwhite and/or Hispanic populations.

c Region within Columbia and Greene counties containing CBGs composed of 100% rural populations, as defined by the US Census Bureau (25).

d Region within Columbia and Greene counties containing CBGs not included in the rural community.

**Table 2:** Mean and Median Number of Cash Registers and Store Hours Open Per Week, by Type of Food Store, Albany, Columbia County, and Greene County, New York, 2003

Type of Food Store^a^	Total	No. of Cash Registers (c*i*)^b^		No. of Hours Open Per Week (h*i*)^b^		Adjustment Weight (w*i*)^b^	

		Mean	Median	Mean	Median	Mean	Median
Super produce^c^	16	9.2	7.0	117.2	105.0	2.9	3.0
Year-round produce^d^	54	1.7	2.0	91.6	84.0	1.2	1.0
Seasonal produce^e^	26	2.0	1.0	42.6	49.0	0.5	0.5
Fruit-for-snack^f^	37	1.4	1.0	113.4	119.0	1.2	1.0
Non-F&V	130	1.6	1.0	104.7	105.0	1.2	1.0
Total food stores	263	2.1	1.0	97.9	98.0	1.2	1.0

Abbreviation: F&V, fruit and vegetable.

a Food store categorized as an F&V store if it stocked at least 2 types of fresh fruit, excluding lemons and limes, and at least 3 types of fresh vegetables, including at least 1 dark-green or orange-colored vegetable.

b w*i* = (1 + nlog c*i* )(h*i* / 98), where w*i* = adjustment weight, c*i* = number of cash registers, and h*i* = business hours per week. A constant of 98 is used for h*i*, if h*i* >98.

c An F&V store defined as a supermarket with a produce department.

d An F&V store defined as a nonsupermarket store that operates year-round and includes grocery stores, convenience stores, and specialty food stores.

e An F&V store that includes seasonal farm (ie, barn) stores, roadside huts and stands, and farmers' markets.

f Defined as stores carrying at least 1 type of ready-to-eat fresh fruit, such as apples, oranges, and bananas. Although these stores did not meet the F&V measure, they were not categorized as non-F&V stores.

**Table 3:** Unadjusted and Weight-Adjusted Fruit and Vegetable Availability, by Store Density Per 10,000 Residents, Albany, Columbia County, and Greene County, New York, 2003

Store Type	Minority Neighborhood^a^	Mixed Neighborhood^b^		Rural Community^c^		Small-Town Community^d^	

	Density	Density	*P* Value	Density	*P* Value	Density	*P* Value
**Unadjusted**							
F&V store^e^, total	4.2	7.3	.19	6.5	.20	6.8	.16
Super produce^f^	0.0	2.0	.02	1.0	.11	1.4	.06
Year-round produce^g^	4.2	3.3	.66	3.7	.73	3.0	.40
Seasonal produce^h^	0.0	2.0	.02	1.8	.03	2.4	.01
Fruit-for-snack store^i^	2.3	2.7	.80	2.9	.62	1.8	.64
Non-F&V store	13.8	9.4	.22	5.8	<.001	8.9	.05
Total food store	20.3	19.4	.84	15.2	.09	17.5	.39
**Weight-adjusted**							
F&V store^e^, total	4.6	11.4	.01	7.8	.10	9.8	.02
Super produce^f^	0.0	7.3	<.001	2.4	.01	4.0	.001
Year-round produce^g^	4.6	4.0	.78	4.4	.90	4.2	.80
Seasonal produce^h^	0.0	0.0	<.99	1.0	.11	1.6	.04
Fruit-for-snack^i^	2.7	2.0	.66	3.4	.60	2.4	.80
Non-F&V store	15.4	12.0	.38	6.5	<.001	10.7	.08
Total food store	22.7	25.4	.59	17.7	.12	22.9	.96

Abbreviations: F&V, fruit and vegetable; CBGs, census block groups.

a Region within the city of Albany containing CBGs composed of ≥50% nonwhite and/or Hispanic populations. Reference category for comparison.

b Region within the city of Albany containing CBGs composed of <50% nonwhite and/or Hispanic populations.

c Region within Columbia and Greene counties containing CBGs composed of 100% rural populations, as defined by the US Census Bureau (25).

d Region within Columbia and Greene counties containing CBGs not included in the rural community.

e Food store categorized as an F&V store if it stocked at least 2 types of fresh fruit, excluding lemons and limes, and at least 3 types of fresh vegetables, including at least 1 dark-green or orange-colored vegetable.

f An F&V store defined as a supermarket with a produce department.

g An F&V store defined as a nonsupermarket store that operates year-round and includes grocery stores, convenience stores, and specialty food stores.

h An F&V store that includes seasonal farm (ie, barn) stores, roadside huts and stands, and farmers' markets.

i Defined as stores carrying at least 1 type of ready-to-eat fresh fruit, such as apples, oranges, and bananas. Although these stores did not meet the F&V measure, they were not categorized as non-F&V stores
